# Nrf2 plays a pivotal role in protection against burn trauma-induced intestinal injury and death

**DOI:** 10.18632/oncotarget.8189

**Published:** 2016-03-18

**Authors:** Zhao Chen, Yiran Zhang, Liang Ma, Yiming Ni, Haige Zhao

**Affiliations:** ^1^ Department of Cardiothoracic Surgery, The First Affiliated Hospital, School of Medicine, Zhejiang University, Hangzhou, Zhejiang, China

**Keywords:** Nrf2, burn trauma, intestinal injury, systemic inflammation, Immunology and Microbiology Section, Immune response, Immunity

## Abstract

Nuclear factor (erythroid-derived 2)–like 2 (NRF2) is a basic leucine zipper transcription factor that principally defends against oxidative stress and also plays a unique role in severe sepsis. However, its contribution to intestinal injury and death after burn trauma is unclear.In this study, wild-type (Nrf2^+/+^) and Nrf2-deficient (Nrf2^−/−^) mice were subjected to 15% or 30% total body surface area burn or sham injury. Survival, systemic inflammation, and gut injury were determined. Nrf2^−/−^ mice were more susceptible to burn-induced intestinal injury, as characterized by increases in damage to the gut structure and in intestinal permeability. This exacerbation was associated with an increase in the intestinal mRNA expression of inflammatory cytokines (interleukin [IL]–6, IL-1B, monocyte chemotactic protein 1, intercellular adhesion molecule, and vascular cell adhesion molecule) and a decrease in the intestinal mRNA expression of Nrf2-regulated genes (NAD(P)H dehydrogenasequinine-1 and glutamate-cysteine ligase modifier subunit). Nrf2-deficient mice also showed a lower survival rate and higher levels of systemic cytokines (IL-6 and IL-1B) and high-mobility group protein B1 than wild-type mice. This study demonstrates for the first time that mice that lack Nrf2 are more susceptible to burn-induced intestinal injury and have more systemic inflammation and a lower survival rate.

## INTRODUCTION

As a common surgical injury, burn trauma often induces a systemic inflammatory response syndrome that can progress to distant organ injury and multiple organ failure [1]. To minimize this damage, efforts have been made to decrease the inflammatory response after burn injury [2]. Previous studies have indicated that gut-derived proinflammatory mediators that spread systemically through the intestinal lymph could be responsible for amplifying the systemic response after severe burn injury [3]. An understanding of the signaling mechanisms that modulate intestinal damage and systemic inflammation may be a key target for the improvement of outcomes after burn injury.

Burns are a common form of traumatic injury that are associated with high mortality and morbidity rates [4]. The effects of oxidative stress in burn injuries have been well documented and are hypothesized to contribute to the development of distant organ injury or failure [5]. In experimental and clinical studies, burn-associated oxidative stress has been confirmed by down-regulation of antioxidant activities and oxidative modifications of proteins and lipids in multiple organs [6]. Cetinkale et al. demonstrated that early intervention with antioxidant therapy significantly restored cell-mediated immunity after severe burn injury [7]. In addition, Zang et al. suggested that the loss of the reactive oxygen species (ROS) defense after severe burn injury may play an important role in cardiac dysfunction [8]. Thus, the short-term inflammation and long-term organ injury caused by burn injury have a particularly close connection to oxidative stress and antioxidative reactions.

It has been demonstrated that severe burn injury induces excess intracellular and extracellular ROS production, which leads to a state of oxidative stress [8]. The antioxidant defense system, in turn, protects cells from oxidative stress by confining the ROS levels and inhibiting a redox-mediated inflammatory response. Specifically, proinflammatory factors such as tumor necrosis factor (TNF)-α, interleukin (IL)-6, and IL-12 generate ROS that can be counteracted by the nuclear factor (erythroid-derived 2)-like 2 (Nrf2) transcription factor, which is believed to be the key transcription factor that is activated by oxidative stress and initiates an antioxidant response. Therefore, the activation of Nrf2 helps to protect cells from oxidative stress, thus inhibiting the redox-mediated inflammatory response [9].

The transcription factor Nrf2 belongs to the cap ‘n’ collar family that contains a conserved basic leucine zipper structure. Nrf2 can protect cells against environmental and oxidative stress [10] and maintains the cellular redox balance by regulating endogenous antioxidants, phase II detoxification enzymes, and other defensive proteins via antioxidant response elements in the promoters of its target gene [11]. Under basal conditions, Nrf2 is held in the cytoplasm by Kelch-like ECH-associated protein 1 (Keap1), ubiquitinated by the Cul3-Keap1 E3 ligase complex, and degraded by the proteasome pathway [12]. Keap1-mediated ubiquitination of Nrf2 is impeded in response to oxidative stress, which results in the stabilization of Nrf2. The level of Nrf2 protein is then elevated, and the transcription factor translocates into the nucleus, where it binds promoters that contain antioxidant response elements, resulting in the elevated expression of a series of antioxidants and cytoprotective genes [13]. Well-characterized Nrf2-dependent genes include heme oxygenase-1 (HO-1), NAD(P)H dehydrogenasequinine-1 (NQO1), glutamate-cysteine ligase (GCL), and glutathione S-transferase A1 [11]. In this role, Nrf2 activity has been shown to be an important disease modifier in many oxidative/inflammatory diseases, such as asthma, sepsis, and pulmonary fibrosis, in which decreased Nrf2 activity exacerbates disease progression [14-16].

A series of studies have clarified the crucial effect of Nrf2 in various experimental models of the anti-inflammatory response. For example, Braun et al. revealed the essential roles of Nrf2 in the control of gene expression and the inflammatory response during skin wound healing [17]. In addition, Thimmulappa et al. reported that Nrf2 is a novel regulator of the innate immune response that dramatically improves the likelihood of survival of experimental sepsis by protecting against dysregulated inflammation [14]. Moreover, Wei et al. demonstrated that Nrf2 can limit the intestinal inflammatory response and reduce gut barrier dysfunction after traumatic brain injury [18]. However, to our knowledge, the role of Nrf2 in the systemic inflammatory response after severe burn injury has not been reported, and the underlying mechanism has not been clarified. Therefore, the purpose of this study was to investigate the role of Nrf2 in the modulation of burn-induced up-regulation of inflammatory mediators.

## RESULTS

### Disruption of Nrf2 causes a drastic increase in lethality after burn injury

**Figure 1:**
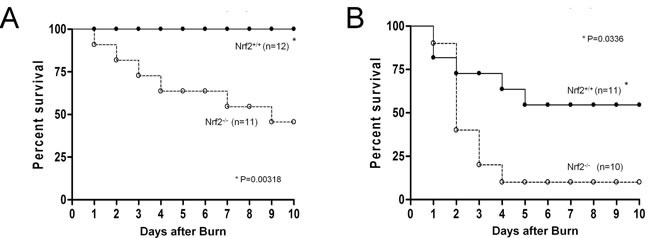
Nrf2 protects from mortality induced by burn injury **A.** Survival curves of Nrf2+/+ and Nrf2−/− mice after 15% TBSA burn injury. **B.** Survival curves of Nrf2+/+ and Nrf2−/− mice after 30% TBSA burn injury. Mortality was monitored every 24 hours for 10 days.

In our previous experiments, Nrf2 was shown to be a critical transcription factor for survival of lethal septic shock [19]. To determine the role of Nrf2 in burn injury, Nrf2^+/+^ and Nrf2^−/−^ mice were subjected to 15% or 30% TBSA burn injury, and their survival was monitored for 10 days. After 15% TBSA burn injury, more than 50% of Nrf2^−/−^ mice died by the tenth day, but no deaths occurred in the Nrf2^+/+^ mice (Figure 1A). After 30% TBSA burn injury, 90% of the Nrf2^−/−^ mice died by the tenth day, whereas only 45.5% of the Nrf2^+/+^ mice died by the same time point (Figure 1B). The survival rate of the Nrf2^−/−^ mice was significantly worse than that of the Nrf2^+/+^ mice after both 15% TBSA and 30% TBSA burn injury (p < 0.05). These data demonstrate a critical role for the Nrf2 pathway in survival after burn injury.

### Nrf2^−/−^ mice display greater systemic inflammation than Nrf2^+/+^ mice

**Figure 2:**
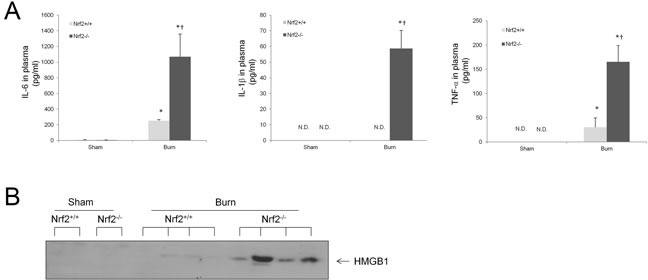
Higher systemic inflammation in Nrf2 ^−/−^ mice subjected to 30% TBSA burn injury compared to Nrf2^+/+^ mice **A.** Plasma IL-6, IL-1β, and TNF-α levels at 24 hours after burn injury. The results represent mean cytokines levels ± SEM (*n* ≥ 4). N.D.: None Detected, *p < 0.05 *vs* Sham, † *p* < 0.05 *vs* Nrf2+/+. **B.** Plasma HMGB1 levels in mice 24 h after burn injury as analyzed by immunoblot. Each band represents levels of HMGB1 in 3 μl plasma of in an individual mouse.

The inflammatory cytokines IL-6, IL-1β, and TNF-α have been well documented to increase mortality in sepsis [19]. To detect the plasma levels of these proinflammatory cytokines after burn injury, plasma from Nrf2^+/+^ and Nrf2^−/−^ mice was collected for ELISA assay 24 h after 30% TBSA burn injury. The plasma levels of IL-6 and TNF-α were significantly elevated after burn injury and were significantly higher in the Nrf2^−/−^ mice than in the Nrf2^+/+^ mice (Figure 2A). The plasma level of IL-1β remained undetectable after burn injury in Nrf2^+/+^ mice, whereas it was elevated significantly after burn injury in Nrf2^−/−^ mice (Figure 2A). HMGB1, another well-characterized pleiotropic cytokine, is a crucial late mediator of mortality in sepsis [20]. Specifically, secretory HMGB1 augments the proinflammatory response and promotes inflammation and tissue injury [21]. Importantly, the plasma levels of HMGB1 were significantly higher in the Nrf2^−/−^ mice than in the Nrf2^+/+^ mice after burn injury (Figure 2B). These data suggest that Nrf2 activity is crucial in the modulation of lethal systemic inflammation after burn injury.

### Nrf2^−/−^ mice exhibit severe intestinal injury after burn injury

**Figure 3:**
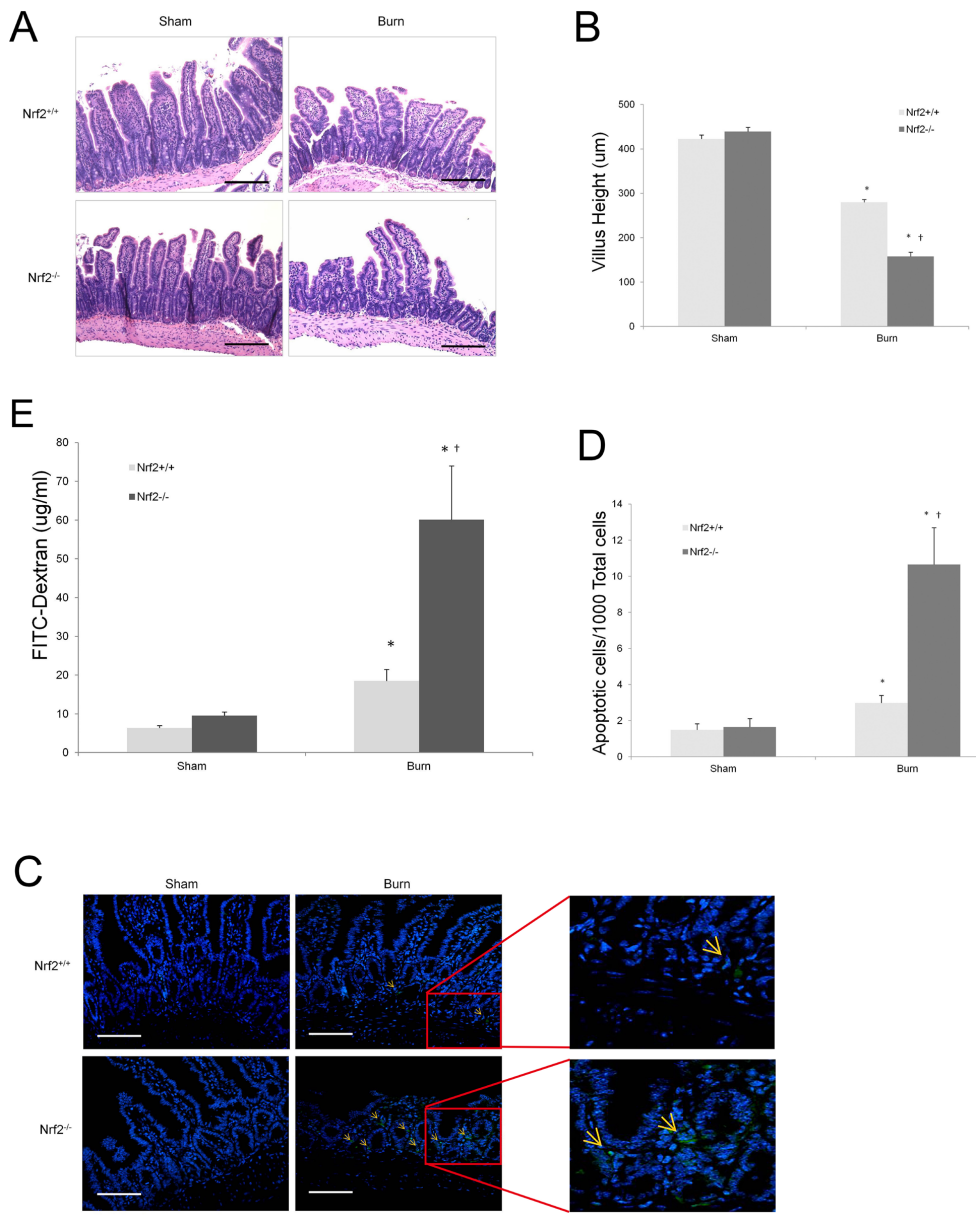
Increased injury on intestines in Nrf2^−/−^ mice after 30% TBSA burn injury **A.** Histopathological analysis of intestines by H&E staining 24 hours after burn injury. Magnification, ×10. Scale bars represent 200 μm. **B.** Changes of villus height after burn injury. Villus height was measured in 5 random fields in each slice. 3 mice were counted in each group. Data are expressed as mean ± SEM. **C.** Apoptotic cells in sections of intestinal tissues were detected by TUNEL assay according to the manufacturer's instructions (green staining). Confocal laser scanning microscopy analyzed samples. Magnification, ×20. Scale bars represent 100 μm. **D.** Quantitative analysis of apoptotic cells. 5 random fields were counted in each slice. 3 mice were counted in each group. Data are expressed as mean ± SEM. **E.** Intestinal permeability was assessed 4h after burn by measuring the systemic plasma concentration of intraluminally injected 4.4-kd FITC-dextran. Data are expressed as mean ± SEM. *p < 0.05 *vs* Sham, †*p* < 0.05 *vs* Nrf2+/+.

The gastrointestinal tract plays a central role in the immune response to burn injury, not only because gut-derived bacteria can cause distant organ injury, but because the gut itself is also known as a cytokine-secreting organ [22]. Thus, we investigated the role of Nrf2 in intestinal injury after burn injury. Sections of proximal ileum from each experimental group were assessed for evidence of histologic injury 24 h after burn injury. The histologic appearance of the intestines of the burned animals demonstrated marked blunting and shortening of the villi, especially in the Nrf2^−/−^ mice (Figure 3A). The villi were also significantly shorter in the Nrf2^−/−^ mice than in the Nrf2^+/+^ mice (157 ± 9.08 μm vs. 279 ± 6.21 μm; p < 0.0001; Figure 3B). Burn injury also led to an increase in the number of TUNEL-positive cells in both Nrf2^+/+^ and Nrf2^−/−^ mice, but this increase was three times greater in the Nrf2^−/−^ mice than in the Nrf2^+/+^ mice (Figure 3C, 3D). Intestinal permeability is a good indicator of the gut barrier function, which is often compromised after major thermal injury [23]. It has been reported that intestinal permeability can increase for up to 5 h after burn injury [24]. Consistent with this finding, in our study, both Nrf2^+/+^ and Nrf2^−/−^ mice showed increased intestinal permeability 4 h after burn injury, but the increase was four times greater in the Nrf2^−/−^ mice than in the Nrf2^+/+^ mice (Figure 3E).

### Intestines of Nrf2^−/−^ mice subjected to burn injury have higher expression of cytokines and chemokines than those of Nrf2^+/+^ mice

**Figure 4:**
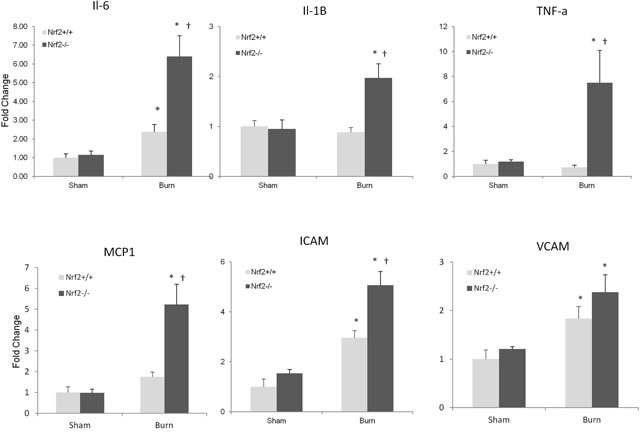
Higher cytokines and chemokines genes expression in intestine of Nrf2^−/−^ mice subjected to 30% TBSA burn injury compared to Nrf2 ^+/+^ mice The mRNA levels of cytokines and chemokines were measured by quantitative PCR (*p* ≥ 4). All genes were normalized by GADPH. Data are expressed as mean ± SEM. *p < 0.05 *vs* Sham, † *p* < 0.05 *vs* Nrf2+/+.

Inflammation of the intestine has been reported to be mediated by proinflammatory cytokines and chemokines, such as IL-6, IL-1β, TNF-α, monocyte chemotactic protein 1, intercellular adhesion molecule (ICAM), and vascular cell adhesion molecule. Therefore, we determined whether the expression of these cytokine and chemokine genes were altered in the intestine after burn injury. We found that burn injury led to an increase in the mRNA expression of IL-6 and ICAM in both Nrf2^+/+^ and Nrf2^−/−^ mice; this increase was significantly greater in the Nrf2^−/−^ mice. Moreover, a dramatic increase in IL-1β, TNF-α, and monocyte chemotactic protein 1 was seen after burn injury in the Nrf2^−/−^ mice, but no significant increase was seen in the Nrf2^+/+^ mice. The mRNA expression of vascular cell adhesion molecule increased significantly after burn injury in both Nrf2^+/+^ and Nrf2^−/−^ mice, but this increase was similar in the Nrf2^+/+^ and Nrf2^−/−^ mice (Figure 4).

### Expression of Nrf2-regulated genes in intestine is increased after burn injury

**Figure 5:**
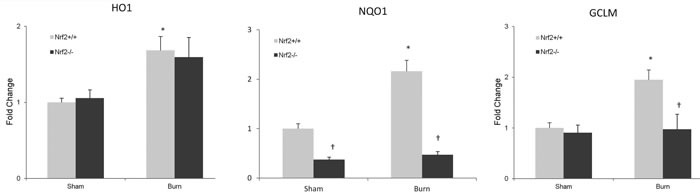
Nrf2-regulated genes expression in intestine after 30% TBSA burn injury The mRNA levels of Nrf2-regulated genes were measured by quantitative PCR (n≥4). All genes were normalized by GADPH. Data are expressed as mean ± SEM. * *p* < 0.05 *vs* Sham, † *p* < 0.05 *vs* Nrf2+/+.

Nrf2 is a cytoprotective transcription factor that regulates antioxidative genes. Therefore, we investigated the expression of Nrf2-regulated genes such as HO-1, NQO1, and GCLM in the intestine. After burn injury, the mRNA expression of NQO1 and GCLM was noticeably increased in the wild-type mice, whereas no change was observed in the Nrf2^−/−^ mice. However, the expression of HO-1 increased in both Nrf2^+/+^ and Nrf2^−/−^ mice, and no significant difference was seen between these two groups (Figure 5).

### Lung inflammation is greater in Nrf2^−/−^ mice after burn injury

**Figure 6:**
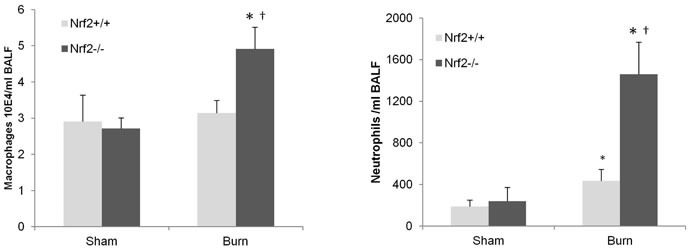
Greater lung inflammation as assessed by BAL assay in Nrf2^−/−^ mice after 30% TBSA burn injury BAL fluid was taken from Nrf2−/− and Nrf2+/+ mice at 8 hours after burn injury. Differential cell counts were performed as mentioned in methods. Data are expressed as mean ± SEM. * *p* < 0.05 *vs* Sham, † *p* < 0.05 *vs* Nrf2+/+.

Consistent with the increased inflammatory response observed in the Nrf2^−/−^ mice, greater lung inflammation was seen in the Nrf2^−/−^ mice after 30% TBSA burn injury as measured by bronchoalveolar lavage fluid assay (Figure 6). The lavage fluid was taken from Nrf2^−/−^ and Nrf2^+/+^ mice 8 h after burn injury. Differential cell counts were performed as previously mentioned [14].

### Lipopolysaccharide-induced cytokine production and nuclear factor kappa B activity are elevated in peritoneal macrophages from Nrf2^−/−^ mice after burn injury

**Figure 7:**
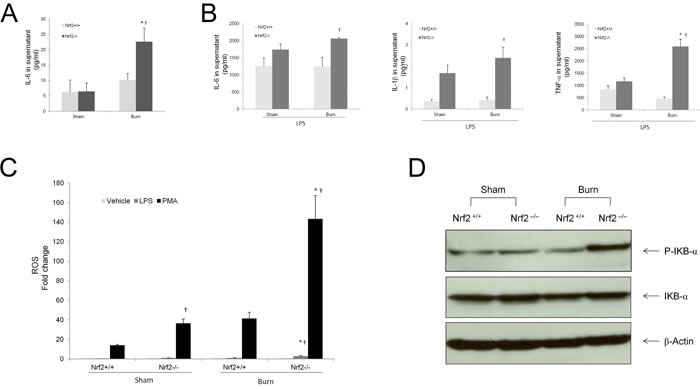
Elevated LPS induced cytokine production and higher NF-kB activity in peritoneal macrophages from Nrf2−/− mice after burn injury **A.** Cytokines production by peritoneal macrophages harvested at 24 hours after 30%TBSA burn injury and then cultured in 2.5×105 cells/ml for 6 hours. TNF-α and IL-1β were not detectable in supernatant. **B.** Cytokines production by peritoneal macrophages cultured with LPS (100ng/ml) for 6 hours. Data are expressed as mean ± SEM. *p < 0.05 *vs* Sham, † *p* < 0.05 *vs* Nrf2+/+. **C.** ROS levels in peritoneal macrophages stimulated with vehicle, LPS (100 ng/ml), or PMA (100ng/ml) for 15 minutes. Each bar is the means ± SEM (*n* = 3) of values presenting the integration of the area under curve expressed in counts. **D.** Burn injury significantly increased the expression of P-IKB-α in macrophages from Nrf2−/− mice, compared with the Nrf2+/+ mice.

Peritoneal macrophages were harvested 24 h after 30% TBSA burn injury and co-cultured with lipopolysaccharide (LPS; 0 and 100 ng/ml) for 6 h. Supernatants were collected for measurement of cytokine production with an ELISA kit. Without LPS challenge, the secretion of IL-6 from peritoneal macrophages in the Nrf2^−/−^ mice increased significantly after burn injury as compared to the Nrf2^+/+^ mice (Figure 7A), whereas the secretion of IL-1β and TNF-α were undetectable in the supernatants of both groups. After LPS challenge, Nrf2 deficiency caused a significant increase in the production of IL-6, IL-1β, and TNF-α by peritoneal macrophages after burn injury (Figure 7B). ROS are linked with cytokine production in sepsis. In agreement with this finding, ROS generation by peritoneal macrophages challenged with phorbol ester showed a dramatic increase in Nrf2^−/−^ mice after burn injury as compared with the Nrf2^+/+^ mice. The same observation was made when peritoneal macrophages were challenged with LPS (Figure 7C). To determine the activity of nuclear factor kappa B (NF-κB), peritoneal macrophages were harvested 2 h after 30% TBSA burn injury and the expression of P-IKB-α and IKB-α were analyzed by immunoblot. Burn injury significantly increased the expression of P-IKB-α in macrophages from the Nrf2^−/−^ mice, but not in those from the Nrf2^+/+^ mice (Figure 7D).

## DISCUSSION

The role of Nrf2 in the anti-inflammatory response has been clarified in various experimental models [14, 17, 18], but its role in the systemic inflammatory response after severe burn injury is still unclear. Our findings show that Nrf2 deficiency in mice leads to a higher mortality rate, an elevated systemic inflammation response, and more severe intestinal injury and lung inflammation after burn injury, and suggest that Nrf2 can decrease inflammatory response after burn injury. These results are consistent with those reported by Jin et al. [18], who studied the role of Nrf2 in the intestinal inflammatory response and gut barrier dysfunction after traumatic brain injury. They also found that Nrf2^−/−^ mice showed significantly higher intestinal levels of NF-κB, proinflammatory cytokines, and ICAM-1 than Nrf2^+/+^ mice after traumatic brain injury, along with higher intestinal permeability and plasma levels of endotoxin. Their data indicate that Nrf2 reduces neutrophil infiltration and decrease the inflammatory response after severe sepsis.

The intestinal barrier can break down after severe burn injury and induce intestinal inflammation, which may act as the source of the systemic inflammatory response [1]. In our study, Nrf2 deficiency led to more severe intestinal injury after burn injury, as evidenced by significant injury to the villi, a greater number of apoptotic cells, and greater intestinal permeability of the distal ileum. Deitch et al. showed that shock-induced intestinal inflammation results in activation of the mesenteric lymphatic system, which contains gut-derived inflammatory mediators that are carried to the systemic circulation via mesenteric lymphatic vessels [25]. Among them, TNF-α, IL-6, and IL-1β, which can be regulated by the activation of NF-κB, have cytotoxic effects that can induce damage to the microvilli, resulting in the destruction of intercellular tight junctions and increased intestinal permeability [26]. Furthermore, an increase in intestinal permeability permits bacterial and endotoxin translocation, which triggers a systemic inflammatory response to release proinflammatory cytokines and mediators, leading to an aggravated systemic inflammatory response syndrome and multiple organ dysfunction syndrome [27, 28]. Our study showed more severe injury to the villi, more apoptotic cells, and greater intestinal permeability in the distal ileum of Nrf2^−/−^ mice than in that of wild-type mice, which strongly supports the notion that the intestine plays an important role in the initial inflammatory response after severe burn injury and that Nrf2 helps protect the gut barrier by reducing inflammation.

Pulmonary injury is common after trauma, and an association between gut ischemia and secondary lung injury has been shown in several model systems. For example, Demling et al. reported that the initial stages of acute lung injury involve large volumes of fluid influx into the lungs within the first 24 h [29]. Krzyzaniak et al. also observed acute lung injury after burn insult as early as 24 h in a mouse model [1], and preclinical models of endotoxin-mediated sepsis [30] and hemorrhagic shock [31] have been reported. In addition, the pulmonary vascular bed, rather than the liver bed, would be the first vascular bed exposed to intestinal lymph because the mesenteric lymph reaches the systemic circulation by emptying into the subclavian vein, which in turn empties into the heart and then directly into the lungs. In our study, we also found more macrophages in the bronchoalveolar lavage fluid of Nrf2^−/−^ mice than in that of Nrf2^+/+^ mice, which could be a consequence and promoter of systemic inflammation.

Some studies have indicated that high rates of Keap1 mutation and Nrf2 overexpression in papillary adenocarcinoma correlated with a poor prognosis and chemotherapy resistance [32]. Gut barrier dysfunction and enhanced translation of NF-κB, TNF-a, IL-1b, IL-6, and ICAM-1 were observed in Nrf2^−/−^ mice after traumatic brain injury [18]. Mutation of Keap1 in the airway epithelium decreased Keap1 protein levels and thus significantly increased Nrf2, which protected Clara cells against oxidative stress ex vivo and attenuated oxidative stress and cigarette smoke-induced inflammation *in vivo* [33]. Pretreatment with an Nrf2 activator increased antioxidant gene expression in the retina, reduced inflammatory mediator expression, and reduced leukocyte adherence to the retinal vasculature after treatment with LPS in Nrf2^+/+^ mice, but it had no effect in Nrf2^−/−^ mice [34]. Overall, these findings are consistent with our results that Nrf2 plays an important role in inflammatory conditions, including that following burn injury.

In this study, we examined the cytokine production capacity of peritoneal macrophages. Macrophages, which exhibit a strong cytokine production capacity, play a critical role in the regulation of immune responses and secretion of inflammatory mediators, including nitric oxide, prostaglandin E2, TNF-α, and IL-6, after activation via LPS stimulation [35]. Studies have shown that the development of macrophage dysfunction, which is characterized by a reduced or diminished ability to phagocytize bacteria and produce cytokines ex vivo, is often associated with severe sepsis [36]. That is to say, cytokine production by macrophages may reflect the severity of the inflammatory response and tissue damage. Our data reveal that cytokine production by peritoneal macrophages was less pronounced in the absence of Nrf2. The LPS-induced production of cytokines (IL-6, IL1-β, and TNF-α) by peritoneal macrophages was significantly greater after burn injury in the absence of Nrf2 than in Nrf2^+/+^ mice. It is believed that this increase in cytokines promoted the severe inflammation and dysfunction of remote organs, such as the liver and lungs. Our data also show that Nrf2 deficiency led to an increase in NF-κB activity in peritoneal macrophages after burn injury, which suggests that crosstalk may exist between the Nrf2 and NF-κB pathways. Neutrophils also play an important role in innate immune response, and excessive recruitment and accumulation of activated neutrophils in the intestine under pathological conditions is associated with mucosal injury [37]. The role of neutrophils in burn trauma-induced intestinal injury needs to be further studied.

According to our findings, we consider Nrf2 to be a feasible therapeutic target to attenuate organ injury induced by acute inflammation after burn injury. Kong et al. reported that the deficiency of Keap1 in myeloid leukocytes resulted in significant enhancement of the Nrf2 pathway and induced a marked reduction in mortality rate, organ injury, circulating levels of inflammatory mediators, and bacteremia compared with wild-type mice after cecal ligation and puncture [19]. Other studies have reported similar conclusions demonstrating that constitutive activation of Nrf2 by means of tissue-specific disruption of Keap1 diminished oxidative stress, apoptosis, and inflammation in the lungs of mice after exposure to cigarette smoke [38]. In contrast, Thimmulappa et al. performed a preclinical study to evaluate the efficacy of triterpenoids (CDDO-Im and CDDO-Me), which could activate the Nrf2 pathway and protect against an LPS-induced inflammatory response in humans [39]. Treatment with triterpenoids increased nuclear accumulation of Nrf2 protein after LPS stimulation, thus significantly inducing Nrf2-regulated antioxidative genes (HO-1, GCLC, GCLM, and NQO1) in peripheral blood mononuclear cells, which resulted in lower expression of inflammatory cytokines (IL-6, TNF-α). Therefore, the activation of Nrf2 after burn injury may be advantageous for diminishing the inflammatory response and reducing dysfunction of vital organs. Future studies are necessary to determine whether an Nrf2 agonist can be therapeutically beneficial for patients with burn injury.

HO-1, GCLM, and NQO1 are target genes of Nrf2, which play an important role in protecting against inflammatory response and oxidative injury. One previous study showed that induction of HO-1 improved impaired intestinal transit after burn injury [40], while the role of GCLM and NQO1 in burn injury have not been studied so far. Our results showed that the expression of NQO1 and GCLM were decreased in the Nrf2 −/− mice, while the expression of HO1 was not. To explain these results, we speculate that the expression of HO1 may be not only regulated by the Nrf2 gene, but also other potential pathways. Further studies are needed to address this issue.

Not only immune cells but also intestinal epithelial cells can be affected by knocking out of Nrf2 gene. Thus, both increase in inflammatory response and death of intestinal epithelial cells might contribute to the increased intestinal injury and death of mice. Our preliminary results suggest that myeloid specific deletion of Nrf2 increases mortality and systemic inflammation after burn injury (Figure S1). Further study focusing on this issue need to be carried out by selectively knocking out Nrf2 gene in different cell types.

In conclusion, this study shows for the first time that Nrf2 plays a pivotal role in protecting against burn-induced intestinal injury and subsequently improves the likelihood of survival by limiting lethal systemic inflammation. Nrf2 deficiency leads to elevated LPS-induced cytokine production and higher NF-κB activity in peritoneal macrophages, which may partially increase the burn-induced intestinal injury. Future studies are needed to determine whether targeting of the Nrf2 signaling pathway can be a potential novel therapeutic strategy for the treatment of burn injury.

## MATERIALS AND METHODS

### Mice

Nrf2-deficient mice (CD-1; Nrf2^−/−^) were obtained in house as previously described [14]. Briefly, a targeting vector was designed to replace the b-Zip region of nrf2 gene, and embryonic stem cells (E14; 25) were electroporated with a linearized targeting vector. Chimeric male mice generated with positive embryonic stem cell clones were mated with ICR female mice and BALB/cA female mice, and germline transmission of the mutant allele in the offspring was verified by Southern blot analysis. All mice were housed under controlled conditions (temperature and humidity) with a 12-h/12-h light/dark cycle.

All procedures were performed according to the *Guide for the Care and Use of Laboratory Animals* published by the National Institutes of Health (publication 86-23, revised 1985). The protocols were approved by the Animal Care and Use Committee of First Affiliated Hospital, School of Medicine, Zhejiang University.

### Burn injury procedure

The mice received a scald burn as previously described [41, 42]. Briefly, the mice were anesthetized with an intraperitoneal injection of ketamine/xylazine, and their dorsal surfaces were shaved. The pretreated mice were then placed in custom-insulated molds that exposed 15% of their total body surface area (TBSA) along the right dorsum. The molds were immersed in a boiling water bath (95°C to 97°C) for 10 to 12 s, and the animals were immediately towel-dried. Using this approach, a burn injury site covering 30% of the TBSA was induced by exposing both the right and left dorsal surfaces. The mice were then resuscitated with 1 ml of physiological saline solution administered by intraperitoneal injection and returned to their cages. For comparison, the sham-injured mice were subjected to identical anesthesia and immersed in lukewarm water, followed by the same resuscitation procedure.

### Cytokine ELISA

IL-1β and TNF-α in plasma and supernatant were measured with enzyme-linked immunosorbent assay (ELISA) kits according to the manufacturer's instructions (eBioscience, San Diego, CA). IL-6 was measured with ELISA kits purchased from R&D Systems (Minneapolis, MN).

### Immunoblot analysis

Antibodies, anti-IKB, and anti-phosphorylated IKB were obtained from Santa Cruz Biotechnology Inc. (Santa Cruz, CA). Anti-high-mobility group protein B1 (HMGB1) was purchased from Abcam Inc. (Cambridge, MA). Immunoblot analysis was performed as previously described [14].

### Quantitative real time polymerase chain reaction

Total RNA was extracted from the intestines with Trizol (Invitrogen, Carlsbad, CA) according to the manufacturer's instructions. Total RNA (1 μg) was used for cDNA synthesis. Quantitative polymerase chain reaction was performed with commercially available probes (Applied Biosystems, Carlsbad, CA). Assays were performed with the ABI 7000 Taqman system (Applied Biosystems). Glyceraldehyde 3-phosphate dehydrogenase was used for normalization.

### Histopathologic evaluation

Segments of distal ileum (at least three per group) were stored in 10% phosphate-buffered saline solution-buffered formalin and embedded in paraffin blocks with an automated processor. The samples were then cut into 5-μm-thick sections, placed onto glass slides, and stained with hematoxylin-eosin (Richard Allen Scientific, MI). Images were obtained at 10× magnification with an Olympus IX70 light microscope. The villus height was measured with software.

### Bronchoalveolar lavage and phenotyping

The experimental mice were killed with an overdose of ketamine/xylazine. The lungs were then aspirated twice with 1 ml of sterile phosphate-buffered saline solution to collect the bronchoalveolar lavage fluid. Cells were counted with a hemocytometer, and differential cell counts were performed on 300 cells with Wright-Giemsa stain (Baxter, Deerfield, IL).

### ROS measurement

The ROS levels were assessed by means of luminol-dependent chemiluminescence as previously described [43].

### Terminal deoxynucleotidyl transferase deoxyuridine triphosphate nick-end labeling assay

The paraffin-embedded sections of intestine were labeled using terminal deoxynucleotidyl transferase deoxyuridine triphosphate nick-end labeling (TUNEL) assay (TdT-FragEL DNA fragmentation detection kit, Calbiochem, San Diego, CA) according to the manufacturer's instructions. The percentage of TUNEL-positive cells per total cell count in each experimental condition was calculated.

### Intestinal permeability assay

The animals underwent *in vivo* intestinal permeability assay 4 h after the burn injury, according to the method previously described by Costantini et al. [44]. Four hours after the burn injury, animals were anesthetized with inhaled isoflurane. A midline laparotomy was performed, followed by location of the cecum and evisceration of a 5-cm segment of the distal ileum with isolation between silk ties. A previously prepared fluorescein isothiocyanate (FITC)-dextran solution (Sigma-Aldrich, St. Louis, MO; 25 mg 4.4 kDa FITC-dextran in 200 μL phosphate-buffered saline solution) was then injected into the lumen of the isolated ileum. The eviscerated intestine was then returned to the abdominal cavity, and the abdominal wall was closed with silk suture. One hour after FITC-dextran injection, blood was collected by cardiac puncture and placed into heparinized Eppendorf tubes for centrifugation at 10,000*g* for 10 min. The plasma was removed and assayed with a SpectraMax M5 fluorescence spectrophotometer (Molecular Devices, Sunnyvale, CA) to determine the concentration of FITC-dextran. A standard curve for the assay was obtained by serial dilution of FITC-dextran in mouse serum.

### Statistical analysis

Survival studies were analyzed with the log-rank test. All other data were analyzed with an unpaired Student's *t* test or U test. Statistical significance was accepted for P values of less than 0.05.

## SUPPLEMENTARY MATERIAL FIGURE


