# Supplementary material for: Nrf2 plays a pivotal role in protection against burn trauma-induced intestinal injury and death
Source: Oncotarget. 2016 Mar 18;7(15):19272–83. doi: 10.18632/oncotarget.8189 (PMC4991382; doi:10.18632/oncotarget.8189)
Supplement: Supplementary file 1 [file oncotarget-07-19272-s001.pdf]

Nrf2 plays a pivotal role in protection against burn trauma-induced intestinal injury and death

Supplementary Material

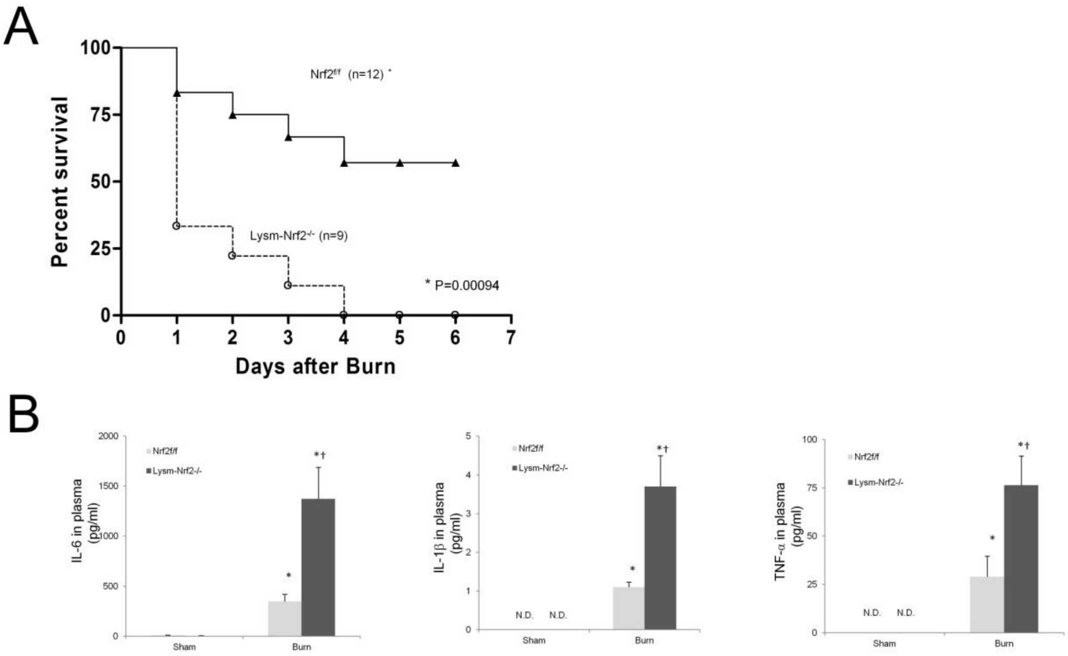

Figure S1. Myeloid specific deletion of Nrf2 increases mortality and systemic inflammation after burn injury. (A) Survival curves of Nrf2$^{f/f}$ and Lysm-Nrf2$^{-/-}$ mice after 30% TBSA burn injury. (B) Plasma IL-6, IL-1β, and TNF-α levels at 24 hours after burn injury. The results represent mean cytokines levels ± SEM (n≥4). N.D.: None Detected, * $p<0.05$ vs Sham, † $p<0.05$ vs Lysm-Nrf2$^{-/-}$.
